# Differentiating Inhibition Selectivity and Binding Affinity of Isocitrate Dehydrogenase 1 Variant Inhibitors

**DOI:** 10.1021/acs.jmedchem.3c00203

**Published:** 2023-03-23

**Authors:** Shuang Liu, Martine Abboud, Victor Mikhailov, Xiao Liu, Raphael Reinbold, Christopher J. Schofield

**Affiliations:** Chemistry Research Laboratory, Department of Chemistry and the Ineos Oxford Institute for Antimicrobial Research, University of Oxford, 12 Mansfield Road, Oxford OX1 3TA, United Kingdom

## Abstract

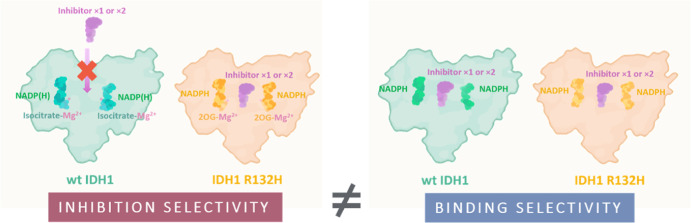

Isocitrate dehydrogenase (IDH) 1/2 gain-of-function variants catalyze the production of the oncometabolite 2-hydroxyglutarate and are validated targets for leukemia treatment. We report binding and inhibition studies on 13 IDH1/2 variant inhibitors, including clinical candidates and drugs, with wild-type (wt) IDH1 and its cancer-associated variant, IDH1 R132H. Interestingly, all the variant inhibitors bind wt IDH1 despite not, or only weakly, inhibiting it. Selective inhibition of the IDH1 R132H variant over wt IDH1 does not principally relate to the affinities of the inhibitors for the resting forms of the enzymes. Rather, the independent binding of Mg^2+^ and 2-oxoglutarate to the IDH1 variant makes the variant more susceptible to allosteric inhibition, compared to the tighter binding of the isocitrate–Mg^2+^ complex substrate to wt IDH1. The results highlight that binding affinity need not correlate with inhibition selectivity and have implications for interpretation of inhibitor screening results with IDH and related enzymes using turnover versus binding assays.

## Introduction

**Figure 1:**
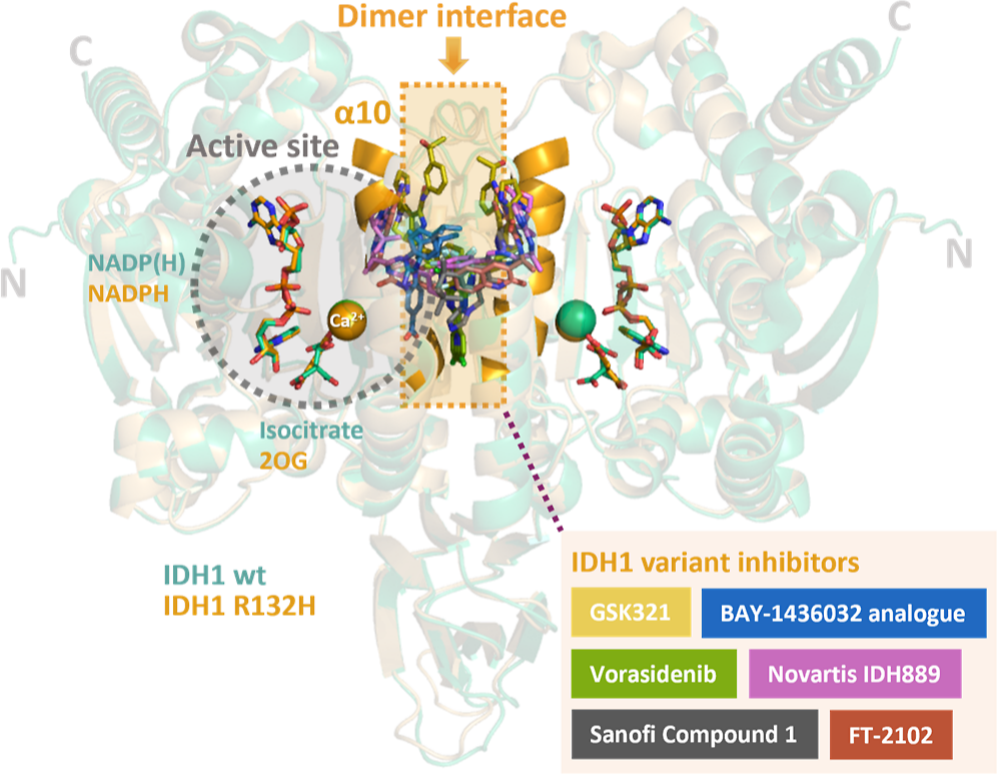
Crystal structure views of IDH1 R132H–inhibitor complexes reveal allosteric binding of multiple inhibitors. Each active site (without inhibitor) contains a substrate (isocitrate for wt IDH1; 2OG for IDH1 R132H), a cosubstrate NADP(H), and a catalytically inhibitory Ca^2+^ which replaces catalytically active Mg^2+^ and is positioned to coordinate to the substrate.^10^ The dimer interface is linked to the active sites of homodimeric IDH1 R132H (PDB 3INM)^11^ and wt IDH1 (PDB 1T0L)^12^ by α10 (N271-G286). Inhibitor binding modes at the dimer interface superimposed onto an IDH1 R132H structure: GSK321 (PDB 5DE1),^6^ an analogue of BAY-1436032 (PDB 5LGE),^7^ Vorasidenib (PDB 6ADG),^13^ Novartis 889 (PDB 5TQH),^14^ Sanofi 1 (PDB 4UMX),^15^ and FT-2102 (PDB 6U4J).^16^

**Scheme 1:**
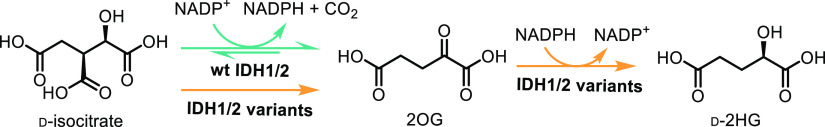
Reactions Catalyzed by Wild-Type IDH1/2 and Their Variants

Mutations in isocitrate dehydrogenase (IDH) 1 and IDH2 genes are found in many cancers, with the most common cancer types associated with IDH1 and IDH2 mutations being gliomas and acute myeloid leukemia (AML), respectively.^1,2^ IDH1 localizes to the cytoplasm, while IDH2 localizes to mitochondria. In addition to wild-type (wt) activity, i.e., NADP^+^-coupled oxidation of isocitrate to 2-oxoglutarate (2OG), the homodimeric IDH1/2 variants have a neomorphic activity, i.e., NADPH-coupled reduction of the wt product 2OG to 2-hydroxyglutarate (2HG) (Scheme 1).^3^ Elevated 2HG levels are proposed to promote tumorigenesis^3^ and suppress antitumor immunity.^4^ Inhibitors of IDH1/2 variants (mIDH1/2) efficiently reduce 2HG levels in vivo, alter cellular metabolism, and are useful for AML treatment, as shown by pioneering studies with Ivosidenib and Enasidenib; mIDH inhibitors are also being explored for treatments of other cancer types, including solid tumors.^5^ Despite their diverse structures and origins, there is extensive crystallographic evidence that most reported mIDH inhibitors do not bind at the active site but instead bind at the IDH homodimer interface which involves an α-helical bundle^6−9^ (Figures 1 and S1).

In most cases, the reported mIDH inhibitors manifest substantial selectivity for inhibition of the IDH variant-catalyzed reaction (2OG to 2HG) over the wt IDH-catalyzed reaction (isocitrate to 2OG), as measured by turnover assays. Recent investigations on the role of Mg^2+^-substrate binding to wt IDH1 and IDH1 R132H have provided a rationalization for the selectivity of two potent allosteric inhibitors, Ivosidenib (AG-120)^17^ and GSK864,^6^ for IDH1 R132H over wt IDH1. The isocitrate–Mg^2+^ complex was shown to be the preferred substrate for human wt IDH1.^10^ By contrast, for IDH1 R132H, separate and weaker binding of 2OG and Mg^2+^ was observed.^10^ α10 of IDH1/2 is both located at the dimer interface and involved in forming the active site (Figure 1). Binding of Ivosidenib and GSK864 on one face of α10 at the dimer interface is proposed to weaken the active site binding of Mg^2+^ and/or the Mg^2+^–substrate complex on the opposite face of α10, in a manner disproportionately affecting 2OG reduction over isocitrate oxidation (Figure 1).^10^

**Figure 2:**
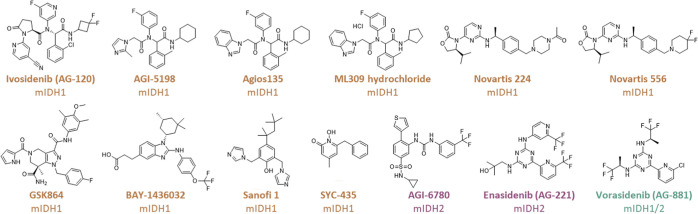
Structures of reported IDH1/2 variant (mIDH1/2) inhibitors profiled in this study. mIDH1 inhibitors: Ivosidenib (AG-120),^17^ AGI-5198,^18^ Agios 135,^15^ ML309 HCl,^19^ Novartis 224,^20^ Novartis 556,^20^ GSK864,^6^ BAY-1436032,^7^ Sanofi 1^15^ and SYC-435;^21^ mIDH2 inhibitors: AGI-6780^22^ and Enasidenib;^23^ and the broad-spectrum mIDH1/2 inhibitor: Vorasidenib (AG-881).^24^

Here, we report studies on wt IDH1 and IDH1 R132H comparing inhibitor binding with the results of turnover assays, the latter of which led to discovery of most of the IDH variant inhibitors. To test the generality of our observations, we investigated 10 mIDH1 inhibitors at different development stages: Ivosidenib,^17^ AGI-5198,^18^ Agios 135,^15^ ML309 HCl,^19^ Novartis 224,^20^ Novartis 556,^20^ GSK864,^6^ BAY-1436032,^7^ Sanofi 1,^15^ and SYC-435;^21^ two mIDH2 inhibitors: AGI-6780^22^ and Enasidenib;^23^ and one broad-spectrum mIDH1/2 inhibitor: Vorasidenib (AG-881)^24^ (Figure 2). The mIDH inhibitors were selected because of their medicinal interest and structural diversity. Ivosidenib (AG-120),^17^ AGI-5198,^18^ Agios135,^15^ and ML309 HCl^19^ were developed in pioneering work by Agios Pharmaceuticals and share a derivatized phenylglycine scaffold. AGI-5198 was a pioneering mIDH1 inhibitor; subsequent optimization led to Ivosidenib which is the first-in-class FDA-approved mIDH1 inhibitor for AML treatment.^17,25^ Novartis 224 and Novartis 556 are analogues of the clinical candidate IDH305 and share a pyrimidine/diazine oxazolidine-2-one core scaffold.^8^ GlaxoSmithKline (GSK) developed GSK864, which is structurally similar to GSK321, but which has improved pharmacokinetic properties;^6^ the relatively low selectivity of the GSK series for mIDHs over wt IDH1 may have hindered its clinical development. The trisubstituted phenyl inhibitor AGI-6780 is the first reported mIDH2 inhibitor,^22^ while Enasidenib (AG-221)^23^ is the first FDA-approved mIDH2 inhibitor for the treatment of AML.^26^ Vorasidenib (AG-881), which like Enasidenib has a triazine core, is the only reported inhibitor that targets both mIDH1 and mIDH2;^24^ it is blood–brain barrier penetrating and is in phase III clinical trial (NCT04164901) for the treatment of low-grade glioma.^24^ BAY-1436032,^7^ Sanofi 1,^15^ and SYC-435^21^ were selected in part because they have distinct scaffolds compared to most mIDH1 inhibitors.

Using recombinant wt IDH1 and the most common IDH variant in gliomas,^27−30^ IDH1 R132H, our biochemical and biophysical studies surprisingly reveal a clear disconnection between inhibitory selectivity and binding affinity of the mIDH inhibitors for wt and R132H IDH1. The results show that inhibitors apparently binding with approximately equal affinity to the resting forms of two closely related enzymes, wt IDH1 and IDH1 R132H, can still manifest selectivity in terms of inhibiting catalysis of only the variant. Selectivity for inhibition of IDH1 R132H over wt IDH1 does not directly relate to the binding affinities of the inhibitors but rather to the higher susceptibility of the variant reaction (2OG to 2HG) to allosteric inhibition involving disruption of active site Mg^2+^ binding, compared to the wt reaction (isocitrate to 2OG).

## Results and Discussion

**Figure 3:**
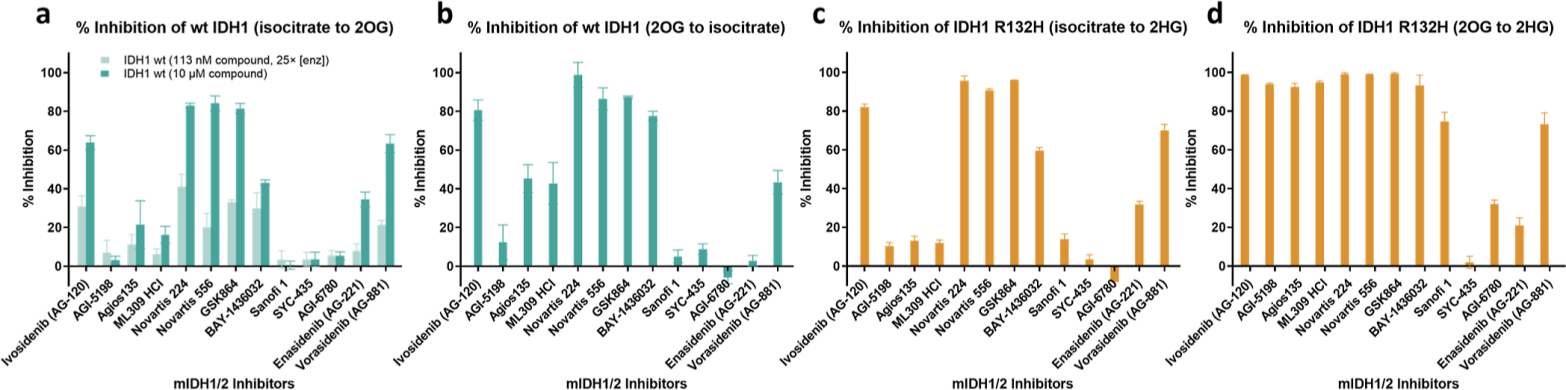
Inhibition of wt IDH1 and IDH1 R132H-catalyzed reactions by mIDH1/2 inhibitors, as measured by absorbance-based turnover assays. % inhibition was calculated by [1—activity of (treated/DMSO control)] × 100%. See the Experimental Section for details. Data are mean ± SD, *n* = 3 technical replicates. (a) % Inhibition of wt IDH1 (4.5 nM)-catalyzed conversion of isocitrate to 2OG by mIDH1/2 inhibitors. Compounds were tested at 113 nM (25× the enzyme concentration) and 10 μM. (b) % Inhibition of wt IDH1 (100 nM)-catalyzed conversion of 2OG to isocitrate by mIDH1/2 inhibitors. Compounds were tested at 2.5 μM (25× the enzyme concentration). (c) % Inhibition of IDH1 R132H (400 nM)-catalyzed conversion of isocitrate to 2HG by mIDH1/2 inhibitors. The 2OG produced from isocitrate oxidation undergoes IDH1 R132H-catalyzed reduction to 2HG. Compounds were tested at 10 μM (25× the enzyme concentration). (d) % Inhibition of IDH1 R132H (400 nM)-catalyzed conversion of 2OG to 2HG by mIDH1/2 inhibitors. Compounds were tested at 10 μM (25× the enzyme concentration).

We first analyzed inhibition of wt IDH1 and IDH1 R132H using the established turnover assay, monitoring changes in NADPH absorbance at 340 nm. In addition to the standard wt IDH1-catalyzed “forward” oxidation of isocitrate to 2OG and IDH1 R132H-catalyzed reduction of 2OG to 2HG, we measured inhibition of the wt IDH1-catalyzed “reverse” conversion of 2OG to isocitrate, as well as IDH1 R132H-catalyzed conversion of isocitrate to 2HG via 2OG^10^ (Figure 3, Scheme 1). IC_50_ values were measured for those showing >50% inhibition of IDH1 at an inhibitor concentration of 10 μM (Table 1, Figure S2). We employed ^1^H NMR (700 MHz) spectroscopy to simultaneously monitor NADPH, NADP^+^, 2OG, and 2HG levels (Figure S3) in order to validate observations from the absorbance assays.

**Table 1:**
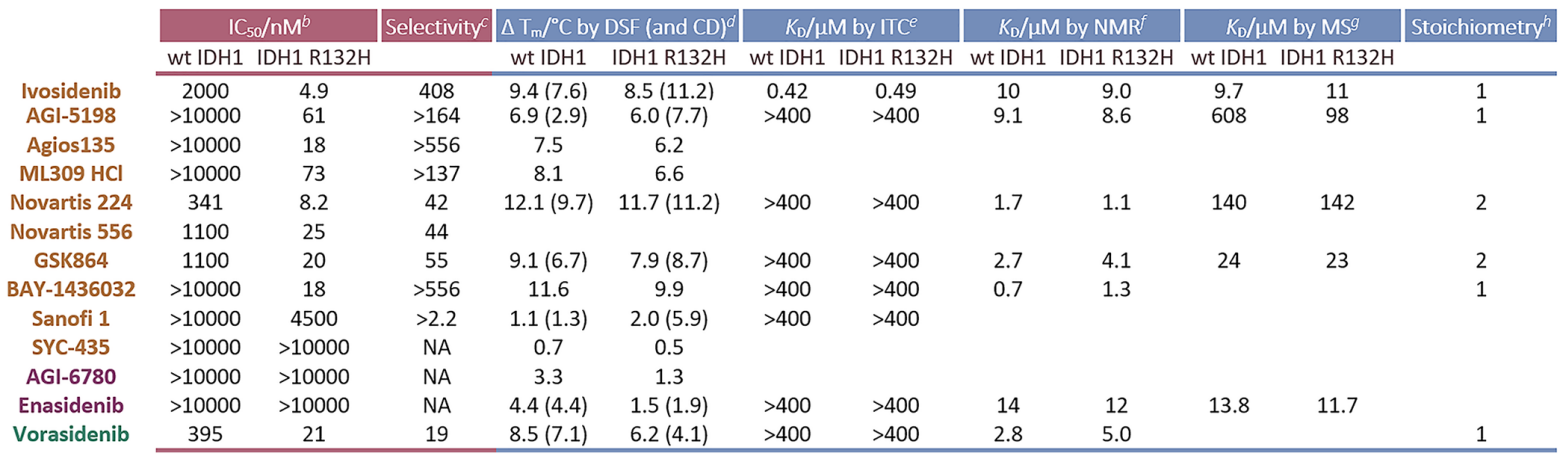
Summary of Biochemical and Biophysical Characterization of IDH1/2 Variant Inhibitors with wt IDH1 and IDH1 R132H^a^

a IDH1 inhibition was measured by absorbance assays. Inhibitor binding was studied by DSF, CD, ITC, NMR, and non-denaturing MS. In general, selectivity was observed for inhibition of catalysis by IDH1 R132H over wt IDH1 but not for inhibitor binding. See the Experimental Section for conditions. IC_50_, half-maximal inhibitory concentration; DSF, differential scanning fluorimetry; CD, circular dichroism; ITC, isothermal titration calorimetry; NMR, nuclear magnetic resonance; and MS, mass spectrometry.

b IC_50_s for wt IDH1 (2 nM)-catalyzed conversion of isocitrate to 2OG and IDH1 R132H (30 nM)-catalyzed conversion of 2OG to 2HG were measured in 100 mM tris–HCl, 10 mM MgCl_2_, 0.005% (v/v) Tween 20, pH 8.0, 0.1 mg/mL BSA, and 0.2 mM DTT. See Figure S2 for IC_50_ plots.

c Selectivity against IDH1 R132H over IDH1 wt, calculated by IC_50_ (IDH1 wt)/IC_50_ (IDH1 R132H).

d The largest change in *T*_m_ of IDH1 (2.5 μM) observed with 3–100 μM inhibitors by DSF is given. See Figure S4 for dose-dependent DSF plots. Values in parentheses are changes in *T*_m_ measured by CD, with 10-fold molar excess of inhibitor (42 μM) relative to IDH (0.2 mg/mL, 4.2 μM). See Figure S5 for CD plots.

e Most inhibitors, except Ivosidenib, manifested no evidence for IDH binding under our ITC conditions. See Figure S6 for ITC plots.

f Determined by line broadening of the inhibitors in ligand-observed ^1^H NMR (700 MHz) spectroscopy. See Figure S9 for *K*_D_ plots.

g Determined by integration of areas under the peaks of IDH and IDH–inhibitor complex from dose-dependent titration of inhibitors with IDH (50 μM). See Figure S7 for non-denaturing MS spectra.

h Number of inhibitors that binds per wt IDH1 or IDH1 R132H dimer, as measured by non-denaturing MS (and by ITC for Ivosidenib) with excess compound(protein/compound = 1:2 to 1:10). See Figures S7 and S8 for non-denaturing MS spectra and Figure 4b for ITC graphs.

For most of the compounds studied, the potencies from the turnover assays were consistent with those reported for individual compounds/series (Table S1). The relative potencies from our work were also consistent with a study comparing mIDH inhibitors by Urban et al.,^31^ though we obtained lower IC_50_s, likely owing to our use of lower IDH concentrations. The extent of inhibition of wt IDH1 and IDH1 R132H by the tested inhibitors at 10 μM for the “forward” oxidation of isocitrate to 2OG is similar (Pearson’s correlation = 0.98; Spearman’s correlation = 0.87) (Figure 3a,c), suggesting conserved general mechanisms for inhibition and possibly catalysis, for the “forward” reaction by wt IDH1 and IDH1 R132H (Scheme 1). This proposal is consistent with the prior observation by NMR spectroscopy that IDH1 R132H catalyzes the “forward” isocitrate oxidation at a comparable rate as wt IDH1, at least under the tested conditions.^10^ Note that in the case of IDH1 R132H-catalyzed conversion of isocitrate to 2HG, the 2OG produced from isocitrate oxidation undergoes IDH1 R132H-catalyzed reduction to 2HG, and it is not possible to identify the effects of inhibitors on the individual reactions under these conditions.

At 10 μM, all the mIDH1 inhibitors fully inhibited IDH1 R132H-catalyzed conversion of 2OG to 2HG, with the exceptions of Sanofi 1 and SYC-435, which showed relatively weak inhibition (Figure 3d). Sanofi 1 was initially reported to have an IC_50_ of 13 nM^15^ but was later reported to have a higher IC_50_ of >13 μM by Urban et al.;^31^ our measured IC_50_ (4.5 μM) is closer to the latter value. SYC-435 has a reported IC_50_ of 190 nM against IDH1 R132H^21^ but under our assay conditions has weak activity (IC_50_ > 10 μM). Vorasidenib, a broad-spectrum mIDH1/2 inhibitor,^24^ shows incomplete inhibition of IDH1 R132H; its IC_50_ plot implies that it does not cause 100% inhibition even at the highest tested concentrations (Figure S2). ^1^H NMR (700 MHz) spectroscopy studies showed that Vorasidenib is a weaker inhibitor of IDH1 R132H compared with Ivosidenib, AGI-5198, Novartis 224, GSK864, or BAY-1436032 (Figure S3).

The mIDH1 inhibitors, including Ivosidenib, Novartis 224, Novartis 556, and GSK864, showed inhibition of wt IDH1 (2 nM) with IC_50_s below 10 μM under our conditions (Table 1). Nevertheless, they retained >40-fold inhibitory selectivity for IDH1 R132H over wt IDH1. The most selective mIDH1 variant inhibitors under our conditions are Agios135 and BAY-1346032, both of which manifest >500-fold inhibitory selectivity for IDH1 R132H over wt IDH1.

To further investigate the mechanisms and selectivity of the inhibitors, we investigated their binding to wt IDH and IDH1 R132H using various biophysical techniques. We first investigated the thermal stabilization of wt IDH1 and IDH1 R132H by mIDH inhibitors using differential scanning fluorimetry (DSF). The extent of thermal stabilization of IDH1 R132H by the mIDH inhibitors was observed to approximately correlate with their potency measured by absorbance assays (Pearson’s correlation = 0.86; Spearman’s correlation = 0.90) (Table 1). Most of the mIDH1 inhibitors thermally stabilize IDH1 R132H in a dose-dependent manner, with the exceptions of Sanofi 1 and SYC-435 (Figure S4), which also did not exhibit strong R132H inhibition in the absorbance assays. Interestingly, although these mIDH inhibitors are, at least partially, selective for IDH1 R132H based on the turnover results (Table 1), they stabilize wt IDH1 to a similar degree as IDH1 R132H (Figures 4a and S4). To the best of our knowledge, this is the first biophysical evidence that multiple allosteric mIDH inhibitors generally appear to be relatively non-selective with respect to binding to wt IDH1 and IDH1 variants. The results of circular dichroism (CD) analyses on thermal stability of IDH1 on inhibitor treatment were generally in agreement with the DSF results (Table 1). In addition, the mIDH1 inhibitors were not observed to cause substantial changes in the secondary structures of wt IDH1 or IDH1 R132H (Figure S5).

**Figure 4:**
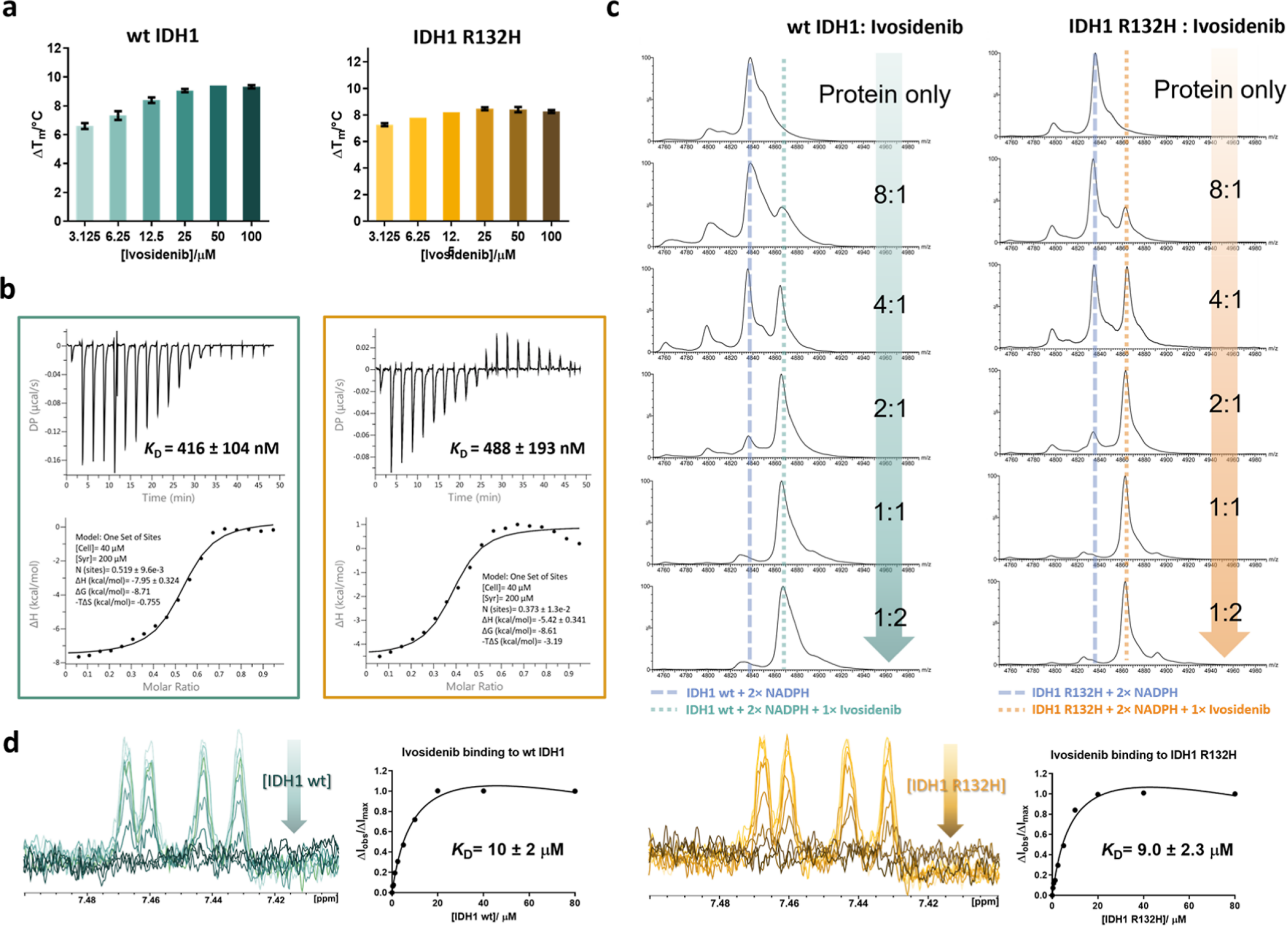
IDH variant inhibitors bind wt IDH1 (teal) and IDH1 R132H (orange) with similar affinities, as exemplified by studies with Ivosidenib using (a) DSF, (b) ITC, (c) non-denaturing MS, and (d) ^1^H NMR (CPMG-edited, 700 MHz) spectroscopy.

Using isothermal titration calorimetry (ITC), under the tested conditions we observed no evidence for tight binding to wt IDH1 and IDH1 R132H for most of the tested inhibitors (Table 1, Figure S6), except for Ivosidenib which is the most potent IDH1 R132H inhibitor (IC_50_ 4.9 nM, Table 1) that binds both wt IDH1 and IDH1 R132H with similar *K*_D_s of 416 and 488 nM, respectively (Figure 4b). The inhibitor-binding stoichiometry, as reflected by ITC (*N* ∼0.5) studies, suggests that one Ivosidenib molecule binds to dimeric IDH1 (Figure 4b).

As most of the mIDH1/2 inhibitors did not cause detectable heat change proteins, as observed by ITC, non-denaturing MS was employed for apparent *K*_D_ measurements by titrating inhibitors against IDH1 proteins (Figures 4c and S7). Consistent with the observations by DSF and ITC, the tested mIDH1/2 inhibitors manifested similar apparent *K*_D_ values for both wt IDH1 and IDH1 R132H (Table 1). Consistent with the ITC results, the non-denaturing MS analyses indicate that Ivosidenib, AGI-5198, and BAY-1436032 bind to wt IDH1 or IDH1 R132H with a stoichiometry of one inhibitor per IDH1 dimer, whereas Novartis 224 and GSK864 bind to IDH1 with the stoichiometry of two inhibitors per IDH1 dimer (Table 1, Figures S7 and S8). The binding stoichiometries determined by non-denaturing MS are consistent with the observations from reported X-ray^6,7,14^ and cryo-EM^32^ structures of the abovementioned inhibitors or their analogues with IDH1 variants. In all cases when wt IDH1 or IDH1 R132H was mixed with the mIDH1/2 inhibitors, we only observed signals for the protein dimer with no evidence for the monomeric form, indicating that inhibition of IDH1 R132H by its inhibitors is not caused by dimer dissociation, at least as reflected in studies with resting enzyme forms.

^1^H NMR (700 MHz) spectroscopy was employed to investigate the binding affinity of mIDH1/2 inhibitors in solution by observing changes in inhibitor signal intensities occurring on protein titration.^33^ Protein binding results in a slower tumbling rate of the inhibitor, faster relaxation of transverse magnetization (shorter *T*_2_), and line broadening.^34^ Consistent with the observations by DSF, ITC, and non-denaturing MS, similar *K*_D_ values/trends in values were observed for the tested inhibitors with respect to binding to wt IDH1 and IDH1 R132H when using NMR (Table 1, Figures 4d and S9). The differences in rank orders of binding affinities for some compounds likely reflect the different assays conditions employed, which may affect conformational dynamics differently, e.g., in gas-phase MS assays versus solution-phase NMR assays.

The addition of MgCl_2_ did not significantly affect the binding affinities of the mIDH1 inhibitors to wt IDH1 and IDH1 R132H, as measured by NMR and ITC studies (Figure S10). This observation may appear inconsistent with the proposed mechanism of inhibition of IDH1 variant inhibitors involving disruption of active site Mg^2+^ binding.^10^ However, manifestation of changes in Mg^2+^ binding that affect catalysis may require formation of enzyme–Mg^2+^–substrate/intermediate complexes. This proposal may in part reflect the different wt/variant selectivities observed in our binding studies with resting forms of the enzymes, where active substrate–metal complexes were excluded to prevent the catalytic turnover, compared to turnover assays which necessarily involve catalytically active substrate–metal complexes.

## Conclusions

The pioneering development of the allosteric mIDH inhibitors by the Agios team^18^ and subsequently multiple other researchers^5,35^ provides an elegant case study of how high-throughput screening can enable development of clinically useful inhibitors working by an unanticipated and conserved allosteric mechanism. The side-by-side inhibition and biophysical studies presented here using a structurally varied set of mIDH inhibitors support the generality of the proposal that mIDH1 inhibitors bind with comparable affinity to both wt IDH1 and IDH1 R132H, despite selectively inhibiting catalysis of 2OG to 2HG by the variant. Thus, the selectivity of the compounds for inhibition of mIDH-catalyzed conversion of 2OG to 2HG over the wt IDH-catalyzed conversion of isocitrate to 2OG does not principally rely on different binding affinities of the inhibitors to the two resting enzymes under our standard assay conditions. Instead, selectivity is due substantially to the higher susceptibility of mIDH-catalyzed 2OG reduction to allosteric inhibition, compared to wt-catalyzed isocitrate oxidation. Mechanistically, such selectivity of the mIDH inhibitors arises, at least in part, because of the tighter binding of the Mg^2+^–isocitrate complex to wt IDH compared to the weaker and (at least, predominantly) independent binding of Mg^2+^ and 2OG to mIDH.^10^ In turn, this reflects the different affinities of isocitrate and 2OG for Mg^2+^. Binding of inhibitors to α10 at the IDH dimer interface disrupts the active site Mg^2+^ binding site which is located on the opposite face of α10. Despite these complexities, the allosteric mIDH inhibitors are clearly active in cells and in vivo, as evidenced by quantitative MS assays revealing a reduction in 2HG levels resulting from inhibition of 2HG producing IDH variants and by their clinical efficacy.^5^

The biophysical results clearly reveal the lack of a linear relationship between the binding affinity and inhibitory potency of the allosteric mIDH inhibitors to wt IDH, that is, the inhibitors bind but do not, or only weakly, inhibit wt IDH1 under the tested concentrations. It may be argued that such a lack of a direct relationship reflects the special nature of the particular enzymes (IDHs/mIDHs) and their reactions. Indeed, a lack of such a relationship is much less likely to be the case for simple active site binding and substrate competitive inhibitors of monomeric enzymes. However, it is proposed that about 30–50% of proteins self-assemble to form complexes with multiple copies of themselves and that most intracellular enzymes operate in multicomponent complexes.^36^ Oligomerization and allosteric ligand binding are established mechanisms of regulating enzyme function; hence, it perhaps should be unsurprising that, at least for some enzymes, appropriately configured high-throughput screens should identify small molecules that operate via allosteric mechanisms, as is the case for the IDH1 R132H inhibitors.

The subtle nature of allosteric inhibition compared to simple substrate competition/active site blockade may mean that initial hits working via allosteric inhibition are more challenging to progress, likely often manifesting more complex structure–activity relationships than active site-binding inhibitors. In this regard, the diversity in structures of mIDH variant inhibitors that apparently all work by the same general mechanism is notable and may in part reflect the dynamic nature of the IDH dimer interface region, including during catalysis. The fact that structurally diverse molecules can bind at the wt IDH/mIDH dimer interface in a manner that sometimes inhibits and sometimes does not also raises the possibility that catalysis by IDH and some other oligomeric enzymes might be regulated in cells in a similar manner by multiple structurally diverse natural small molecules (in vivo allosteric regulation of oligomeric enzymes is well precedented^37^).

Most of the mIDH inhibitors in clinical development likely have their origins in hits from high-throughput screens under fixed biochemical turnover assay conditions. Given that resistance has been reported to the allosterically binding mIDH inhibitors,^38,39^ consideration should be given to the possibility that different types of mIDH inhibitors might be identified by employing different screening assay conditions, e.g., using more than one Mg^2+^ concentrations, or by using binding-based approaches such as DNA-encoded library or peptide library screens^40,41^ with the dimer interface blocked by a known inhibitor, aiming to identify active site rather than dimer interface-binding inhibitors. It may also be possible to identify allosterically binding mIDH modulators that both inhibit the reduction of 2OG to 2HG and promote the oxidation of isocitrate to 2OG.

The combined medicinal chemistry, structural and mechanistic studies on mIDH selective inhibitors highlight the likelihood of considerable scope to identify compounds that modulate the functions of oligomeric biomacromolecules by empirical high-throughput screening involving appropriate turnover and binding assays. Importantly, the results also imply that at least for complex enzyme systems, care should be taken in assuming binding affinity correlates with functional inhibition of enzyme catalysis.

## Experimental Section

### IDH1/2 Variant Inhibitors

Chemicals were used as purchased and were >95% pure by HPLC according to the commercial suppliers. Ivosidenib (AG-120), AGI-5198, Agios 135 (HY-12475), Novartis 224 (HY-18717), Novartis 556 (HY-13972), Vorasidenib (AG-881), BAY-1436032, AGI-6780, and Enasidenib (AG-221) were purchased from MedChem Express. ML309 hydrochloride, GSK864, and Sanofi 1 (SML1430) were purchased from Sigma-Aldrich. SYC-435 (TC-E 5008) was purchased from Bio-Techne.

### Recombinant IDH1 Preparation

Recombinant homodimeric wt IDH1 and IDH1 R132H were produced in high purity from *Escherichia coli* BL21(DE3) pLyS cells as previously described.^10^

### Absorbance Assays

IDH1 activities were measured spectrophotometrically by monitoring changes in NADPH absorbance at 340 nm.^10^ Reactions were monitored at 25 °C continuously for at least 15 min (see details below) using a PHERAstar FS Microplate Reader (BMG Labtech). Percentage inhibition and IC_50_ determinations of compounds for IDH1 were measured in 96-well half area clear microtiter plates (Greiner Bio-One 675001) in a final volume of 100 μL. The difference in absorbance, Δ*A*_340_, in the linear range of the reaction profile was converted to % residual activity using a DMSO control as the 100% residual activity reference. The % inhibition was calculated by (1—activity with inhibitor/activity with DMSO control) ×100%. Experiments were performed in (at least) triplicate. The standard assay buffer for IDH1 consists of 100 mM tris–HCl, 10 mM MgCl_2_, 0.005% (v/v) Tween 20, pH 8.0, 0.2 mM DTT, and 0.1 mg/mL BSA. In general, compounds [25 μL, 0.4% (v/v) DMSO] were pre-incubated with IDH1 (25 μL) for 12 min, followed by addition of dl-isocitrate/2OG (25 μL) and NADP^+^/NADPH (25 μL) to initiate the reaction. The substrate/cofactor concentrations were optimized by determining *K*_M_ values for substrates/cofactors as described.^10^ Standard reaction mixtures contained

(a) 2 nM (for IC_50_ measurements, monitoring for 60 min) or 4.5 nM (for percentage inhibition, monitoring for 15 min) wt IDH1, 150 μM dl-isocitrate and 75 μM NADP^+^ for conversion of isocitrate to 2OG, catalyzed by wt IDH1;
(b) 100 nM wt IDH1, 15 mM 2OG, 500 μM NADPH, 100 mM NAHCO_3_ for conversion of 2OG to isocitrate, catalyzed by wt IDH1 (monitoring for 60 min);
(c) 400 nM IDH1 R132H, 1.5 mM dl-isocitrate, and 75 μM NADP^+^ for conversion of isocitrate to 2OG, catalyzed by IDH1 R132H (monitoring for 60 min);
(d) 30 nM (for IC_50_ measurements, monitoring for 60 min) or 400 nM (for percentage inhibition, monitoring for 15 min) IDH1 R132H, 1.5 mM 2OG and 50 μM NADPH for conversion of 2OG to 2HG, catalyzed by IDH1 R132H.

### Differential Scanning Fluorimetry Studies

Thermal shift experiments were carried out using Sypro Orange and a CFX96 Touch Real-Time PCR Detection System (Bio-Rad) machine.^42^ 96-well white PCR plates were used, in which each well contained 20 μL of 2.5 μM IDH1 with 5× Sypro Orange. The standard assay buffer contains 50 mM tris–HCl at pH 7.5. Various concentrations of compounds (1% DMSO final) were mixed with IDH1 for thermal stabilization studies. Samples in triplicate were subjected to temperature increases from 20 to 95 °C at 0.2 °C min^–1^. Bio-Rad CFX Manager was used to determine the melting temperature, *T*_m_.

### Circular Dichroism Studies

CD spectra were acquired using a Chirascan CD spectrometer (Applied Photophysics) with a Peltier temperature-control cell holder. Experiments were carried out in a 0.1 cm path length quartz cuvette at 20 °C. IDH1 was buffer exchanged into an assay buffer consisting of 10 mM sodium phosphate (chloride ion free), pH 7.5, using a Micro Bio-Spin 6 column (Bio-Rad 732-6221) according to the manufacturer’s protocol. The protein stock solution was diluted with the assay buffer to give a final concentration of 0.2 mg/mL (4.2 μM), and the compound was added (10 mM in MeOH, 1.68 μL) to a final concentration of 42 μM in 400 μL of total volume. A blank sample containing 0.42% (v/v) MeOH was measured. Data were collected in triplicates continuously over wavelengths between 185 and 260 nm with a bandwidth of 1.0 nm in 0.5 nm steps and referenced to the assay buffer. Spectra were averaged (*n* = 3) and smoothed (window size = 7) using the Savitzky–Golay filter. The mean residue ellipticity (MRE) was calculated according to the formula
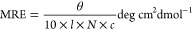
where θ is the degree of ellipticity, *N* is the number of amino acids, *c* is the concentration (mol/L), and *l* is the path length (cm).

For melting temperature determination using CD, the wavelength was fixed at 215 nm, and the temperature gradually increased from 10 to 80 °C at a rate of 1 °C/min. Measurements were conducted in 2 °C increments. The melting temperature was calculated using a Boltzmann sigmoidal model in GraphPad Prism v5.04.

### Isothermal Titration Calorimetry Studies

ITC was conducted using a Malvern MicroCal PEAQ-ITC Automated machine (Malvern Panalytical). In general, IDH1 was prepared in 50 mM tris–HCl, pH 7.5, and the compounds were dissolved in the same buffer. 400 μL of cell solution containing 40 μM IDH1 [4% (v/v) DMSO] and 200 μL of the titrant containing 200 or 400 μM compound [2% or 4% (v/v) DMSO] were transferred to an ITC plate (Malvern Panalytical WEL020854-010). Titrations were conducted at 25 °C with an initial delay of 60 s and a stir speed of 750 rpm. The injection setup consisted of 19 injections, with 1 initial injection of 0.4 μL and 18 subsequent injections of 2.0 μL each with 150 s between the injections. Thermodynamic parameters were obtained using MicroCal PEAQ-ITC analysis software.

### Nuclear Magnetic Resonance Studies

Nuclear magnetic resonance (NMR) reaction monitoring was performed at 298 K using a Bruker AVIII 700 MHz NMR spectrometer with a 5 mm inverse triple-carbon-inverse (TCI) cryoprobe in 5 mm tubes (Norell). Data were recorded with a relaxation delay of 2 s and 32 scans, employing a pulse sequence with water suppression (^1^H excitation sculpting suppression with perfect-echo using a 2 ms Sinc selective inversion pulse). There is a time delay of 184 s between each acquired spectrum over the time-course experiment. Conversion of 2OG to 2HG catalyzed by IDH1 R132H was monitored using 500 μL of 1 μM IDH, 20 μM compound [0.4% (v/v) DMSO, 10 min pre-incubation with IDH], 10 mM MgCl_2_, 1.5 mM NADPH, and 1.5 mM 2OG, in 50 mM tris-D_11_-HCl, pH 7.5 in 90% H_2_O/10% D_2_O (v/v). Data were processed with TopSpin 3.6.1 software. Signals corresponding to NADPH, NADP^+^, 2OG, and 2HG at each time point over a total of 2 to 3 h were integrated, converted to concentrations, and plotted against time. The difference in concentrations in the linear range of the reaction profile was converted to % residual activity with the DMSO control defined as the 100% residual activity reference. The % inhibition was calculated by: (1—activity with inhibitor/activity with DMSO control) ×100%.

Ligand-observed NMR binding studies were performed at 298 K using a Bruker AVIII 700 MHz NMR spectrometer with a 5 mm inverse TCI cryoprobe in 3 mm MATCH tubes (Cortectnet). ^1^H Carr–Purcell–Meiboom–Gill (CPMG) NMR experiments used the PROJECT-CPMG sequence^43^ with τ delays of 0.5 ms and a total filter time (τ × 4 × *N*) of 40 ms. Additional solvent presaturation was applied for 2 s. Samples contained 160 μL of 10 μM compound (0.2% DMSO) in 50 mM tris-D_11_-HCl, pH 7.5 in 90% H_2_O/10% D_2_O (v/v). IDH (1.4 mM) was successively added to the compound from 0.3125 μM to ≥20 μM (final protein concentration) in two-fold increments until compound signals were completely broadened. 200 or 400 scans per IDH concentration were recorded, and signals corresponding to the compound were integrated and processed using TopSpin 3.6.1 software. Graphs of Δ*I*_obs_/Δ*I*_max_ against [protein]/μM were fitted using the binding-saturation (one site-total) model in GraphPad Prism v5.04 to obtain *K*_D_s.
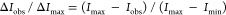
where *I*_max_ is the maximum inhibitor peak integral observed when no protein is added, *I*_obs_ is the observed inhibitor peak integral at a particular protein concentration, and *I*_min_ is the minimum inhibitor peak integral (typically ∼0) when the binding is fully saturated.

### Non-denaturing Protein Mass Spectrometry Studies

Non-denaturing mass spectra were obtained using a quadrupole time-of-flight mass spectrometer (Synapt HD MS, Waters, Manchester, UK).^44,45^ IDH1 stock solutions were exchanged into 200 mM ammonium acetate, pH 7.5 using a Micro Bio-Spin 6 column (Bio-Rad 732-6221). Protein concentrations were measured using a Nanodrop spectrometer (Thermo Scientific NanoDrop One). Compounds were dissolved in MeOH to avoid DMSO interference with mass spectra. IDH1 (20 μL of 50 μM final) and the compound of interest (1–4% (v/v) MeOH) in 200 mM ammonium acetate at pH 7.5 were pipetted into wells of a sample plate of an automated chip-based nano-electrospray ion source (TriVersa Nanomate, Advion, Ithaka, NY, USA). Compounds were serially diluted in buffer and mixed with IDH1 diluted in buffer. The mixture was sprayed through a nozzle in a Nanomate chip at a spray voltage of 1.8–2.0 kV (spray backing gas pressure 0.8–1.0 psi, inlet pressure 3.8 mbar). The sample and extractor cone voltages were 100 and 5.2 V, respectively; no in-source dissociation of the protein dimers was observed at these voltages. Data collection and analyses used Waters MassLynx software. Mass spectra were calibrated externally using CsI solution. *K*_D_ values were calculated using eq 2 as reported^46^ at each compound concentration and are reported as an average across multiple concentrations. For inhibitors with a binding stoichiometry of two inhibitors/IDH dimer, the inhibitor-bound protein peak intensity used was a sum of first and second inhibitor-bound protein peak intensities. Data for the IDH1 dimer *m*/*z* = 20^+^ charge state are shown. For clarity, only 2× NADPH bound protein peaks are annotated.

## Supporting Information Available

Supplementary Figures S1−S10 and their associated references, including: Crystal structure views of dimeric IDH2 R140Q–inhibitor complexes; IC_50_ plots for mIDH1/2 inhibitors with wt IDH1 and IDH1 R132H; inhibition of IDH1 R132H-catalyzed conversion of 2OG to 2HG by mIDH1/2 inhibitors, as monitored by NMR spectroscopy; summary of measured and reported IC50s for IDH1/2 variant inhibitors with wt IDH1 and IDH1 R132H; and DSF, CD, ITC, non-denaturing MS, and NMR analyses of mIDH1/2 inhibitors binding to wt IDH1 and IDH1 R132H (PDF)

## Abbreviations

2HG: 2-hydroxyglutarate
2OG: 2-oxoglutarate
AML: acute myeloid leukemia
CD: circular dichroism
CPMG: Carr–Purcell–Meiboom–Gill
DSF: differential scanning fluorimetry
IDH: isocitrate dehydrogenase
ITC: isothermal titration calorimetry;
*K*_D_: dissociation constant
mIDH: isocitrate dehydrogenase variant or mutant
SD: standard deviation
qTOF: quadrupole time-of-flight
TCI: triple-carbon-inverse
wt: wild-type
