# Supplementary material for: Differentiating Inhibition Selectivity and Binding Affinity of Isocitrate Dehydrogenase 1 Variant Inhibitors
Source: J Med Chem. 2023 Mar 23;66(7):5279–88. doi: 10.1021/acs.jmedchem.3c00203 (PMC10108345; doi:10.1021/acs.jmedchem.3c00203)
Supplement: Supplementary file 1 — jm3c00203_si_001.pdf [file jm3c00203_si_001.pdf]

SUPPORTING INFORMATION

Differentiating inhibition selectivity and binding affinity of isocitrate dehydrogenase 1 variant inhibitors

Shuang Liu, Martine Abboud, Victor Mikhailov, Xiao Liu, Raphael Reinbold, and Christopher J. Schofield*

Chemistry Research Laboratory, Department of Chemistry and the Ineos Oxford Institute for Antimicrobial Research, University of Oxford, 12 Mansfield Road, Oxford OX1 3TA, United Kingdom.

*Correspondence to Christopher J. Schofield: christopher.schofield@chem.ox.ac.uk

Table of Contents

SUPPLEMENTARY FIGURES (S1 – S10) 2

SUPPLEMENTARY REFERENCES 11

SUPPLEMENTARY FIGURES

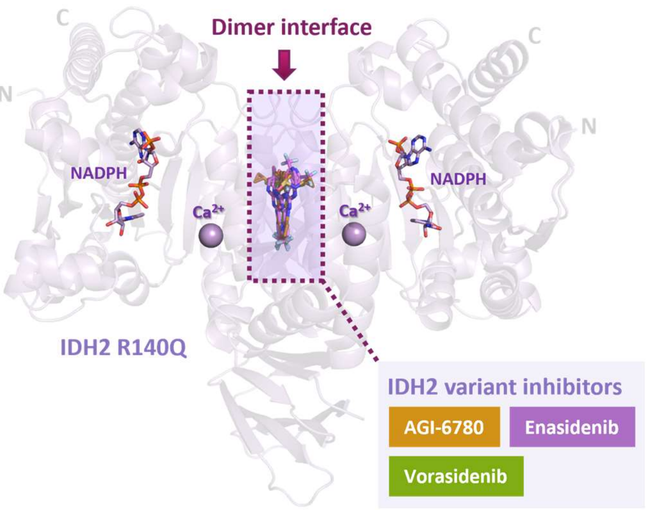

Figure S1. Crystal structure views of dimeric IDH2 R140Q-inhibitor complexes. As observed with mIDH1, IDH2 variant inhibitors bind at the dimer interface of IDH2 R140Q. Each monomer active site (in the presence of inhibitor) contains a cosubstrate (NADPH) and the inhibitory Ca^{2+}. The structure shown is of IDH2 R140Q (semi-transparent purple) in complex with AGI-6780 (orange, PDB 4JA8)[1], superimposed onto which are the binding modes of Enasidenib (lilac, PDB 5I96)[2] and Vorasidenib (green, PDB 6ADI)[3].

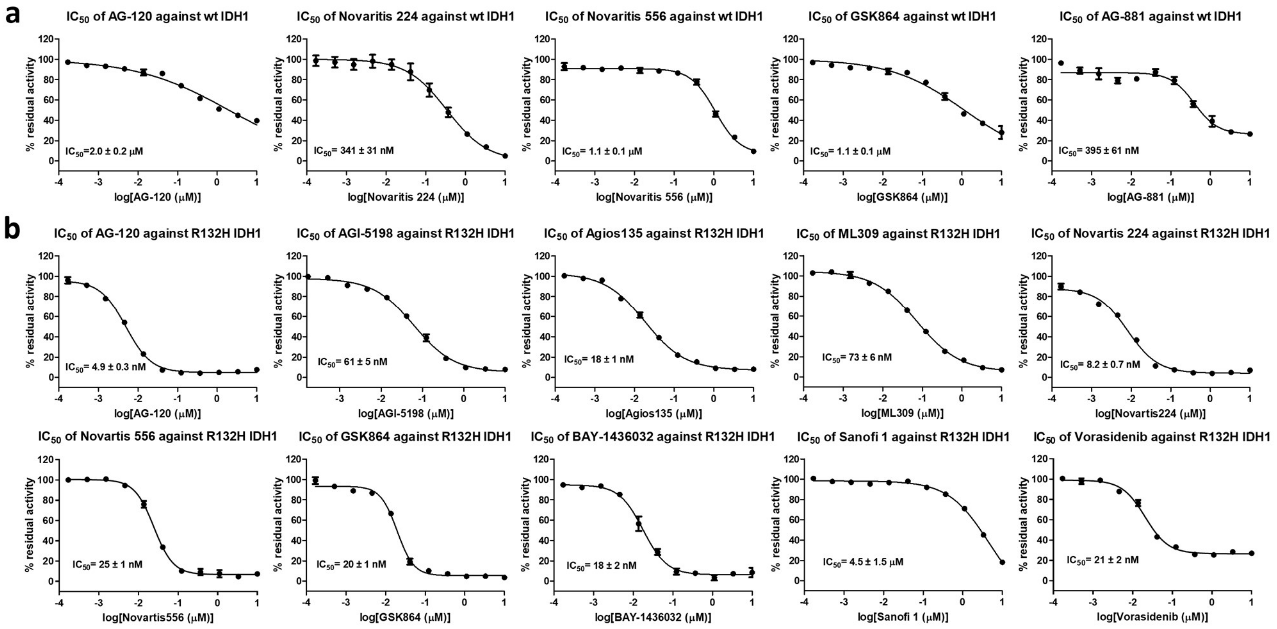

Figure S2. IC_{50} plots for mIDH1/2 inhibitors with wt IDH1 (2 nM) and IDH1 R132H (30 nM), as measured by absorbance assays. Inhibitors were serially diluted 3-fold from 10 μM. See the Experimental Section for details. Data are mean ± SD, n= 3 technical replicates.

a. IC_{50} plots of mIDH1/2 inhibitors against wt IDH1 (2 nM)-catalysed conversion of isocitrate to 2OG.

b. IC_{50} plots of mIDH1/2 inhibitors against IDH1 R132H (30 nM)-catalysed conversion of 2OG to 2HG.

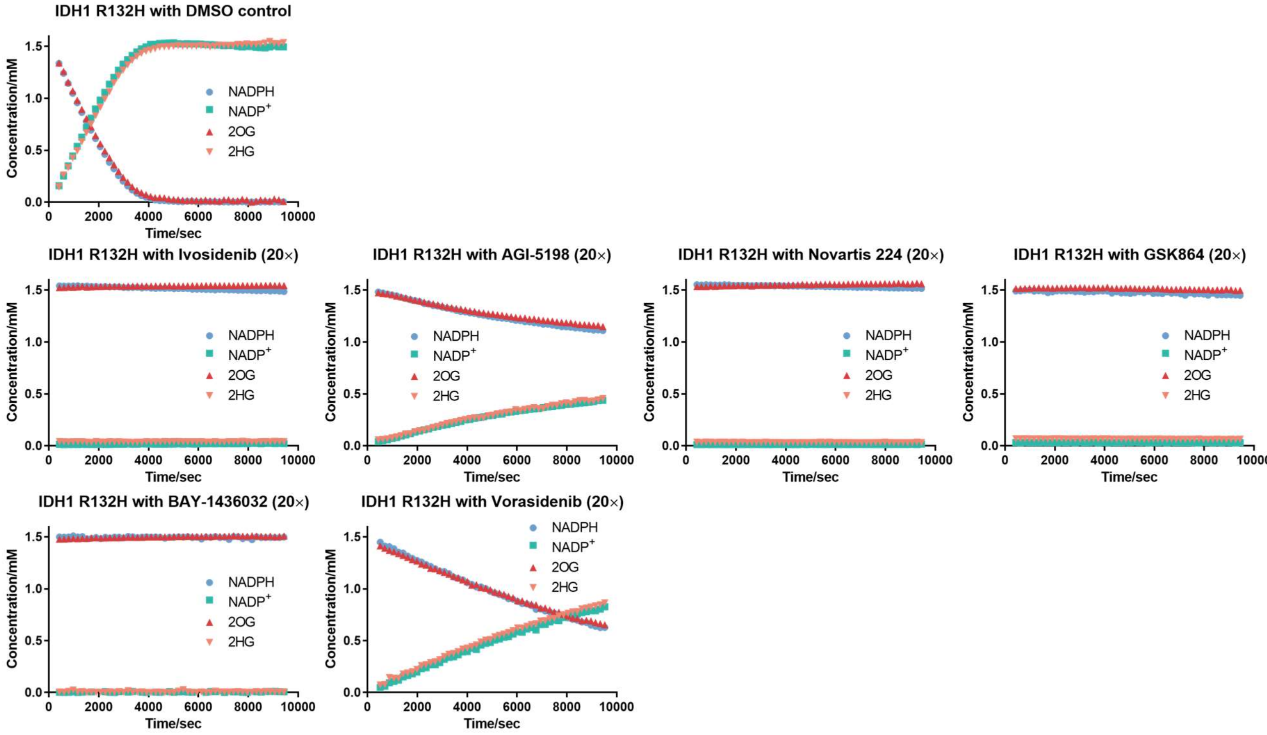

Figure S3. Inhibition of IDH1 R132H catalysed conversion of 2OG to 2HG by mIDH1/2 inhibitors, as monitored by ^{1}H NMR (700 MHz) spectroscopy. The conversion of 2OG and NADPH to 2HG and $NADP^+$, as catalysed by IDH1 R132H in the presence of a DMSO control [0.4% (v/v)], Ivosidenib, AGI-5198, Novartis 224, GSK864, BAY-1436032, or Vorasidenib. The changes in NADPH levels are coupled to changes in levels of 2OG, 2HG and $NADP^+$. Most compounds fully inhibited IDH1 R132H (over 2.5 h), with weaker inhibition being observed for AGI-5198 and Vorasidenib. Assay mixture: 1 μM IDH1 R132H, 20 μM compound [0.4% (v/v) DMSO], 10 mM $MgCl_2$, 1.5 mM NADPH, and 1.5 mM 2OG, in 50 mM Tris-D_{11}-HCl, pH 7.5 in 90% H_2O/10% D_2O (v/v). See the Experimental Section for details.

	IC_{50}/nM (measured)		IC_{50}/nM (reported)	
	wt IDH1	IDH1 R132H	wt IDH1	IDH1 R132H
Ivosidenib	2000	4.9	24/71[4]; 4260[5]	12[4]; 40[5]
AGI-5198	>10000	61	>100000[6]; >30000[5]	70[6]; 390[5]
Agios135	>10000	18	1998[7]; 15600[5]	42[7]; 380[5]
ML309 HCl	>10000	73	36000[8]; 20900[5]	96[8]; 340[5]
Novartis 224	341	8.2	3870[5]	17[9]; 130[5]
Novartis 556	1100	25	10500[5]	<72[9]; 140[5]
GSK864	1100	20	466.5[10]; 2740[5]	15.2[10]; 160[5]
BAY-1436032	>10000	18	20000[11]	15[11]
Sanofi 1	>10000	4500	>10000[7]; >30000[5]	13[7]; 13400[5]
SYC-435	>10000	>10000	12300[12]	190[12]
AGI-6780	>10000	>10000	>100000[1]; >30000[5]	11000[1]; 14400[5]
Enasidenib	>10000	>10000	450/1120[2]; 15000[5]	48/78[2]; 4950[13];
Vorasidenib	395	21	4/190[13]	6/8[13]; 31.9[3]

Table S1. Summary of measured and reported IC_{50}s for IDH1/2 variant inhibitors with wt IDH1 (2 nM) and IDH1 R132H (30 nM). For reported IC_{50} values, "/" refers to measurements from different incubation times from the same study, while ";" separates values from different studies.

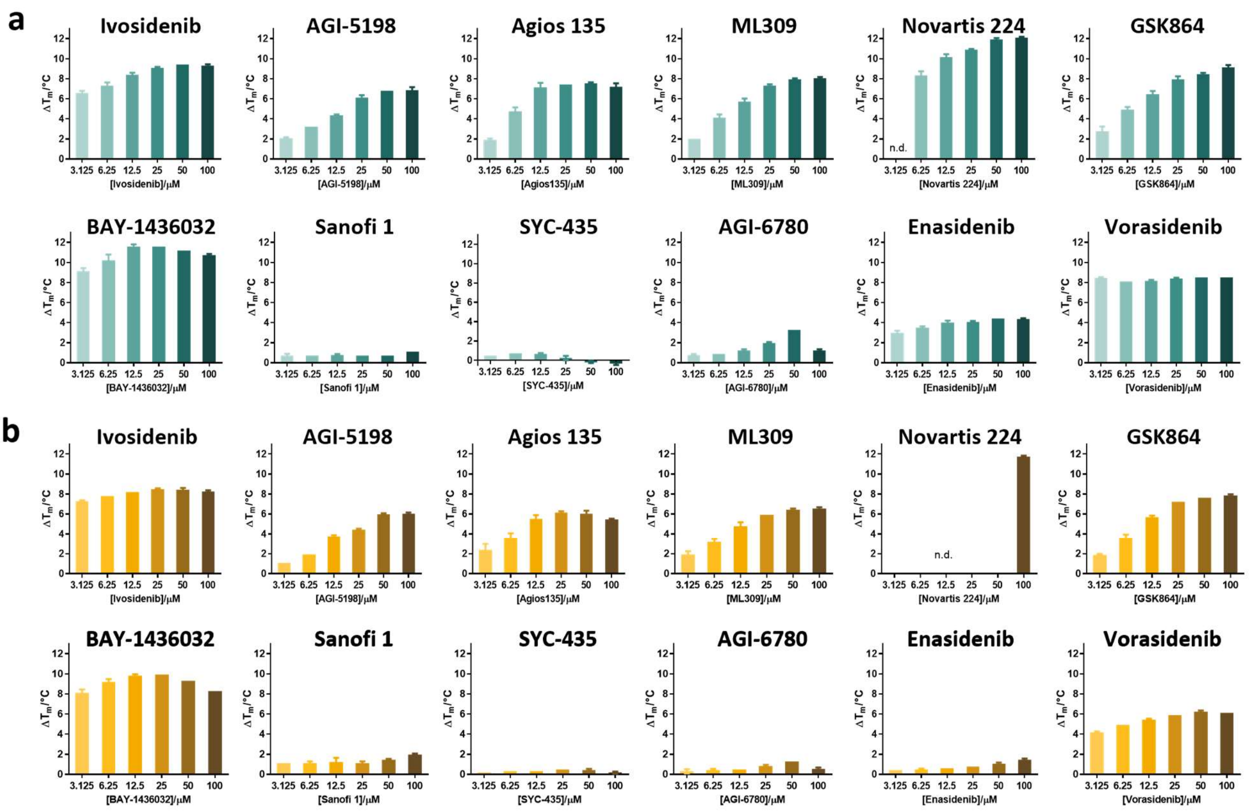

Figure S4. Dose-dependent thermal stabilisation of wt IDH1 and IDH1 R132H by mIDH1/2 inhibitors (3.125–100 μM), in 50 mM Tris-HCl, pH 7.5, as measured by DSF. Data are mean ± SD, n= 3 technical replicates. See the Experimental Section for details. N.d., not determined, as the compound interferes with the fluorescence signals.

a. DSF results for wt IDH1 with mIDH1/2 inhibitors; wt IDH1 is apparently stabilized by all tested mIDH1 and broad-spectrum mIDH1/2 inhibitors, except for Sanofi 1 and SYC-435. mIDH2 inhibitors (AGI-6780 and Enasidenib) weakly stabilise wt IDH1.
b. DSF results for IDH1 R132H with mIDH1/2 inhibitors; IDH1 R132H is apparently stabilized by all tested mIDH1 and broad-spectrum mIDH1/2 inhibitors, except for Sanofi 1 and SYC-435.

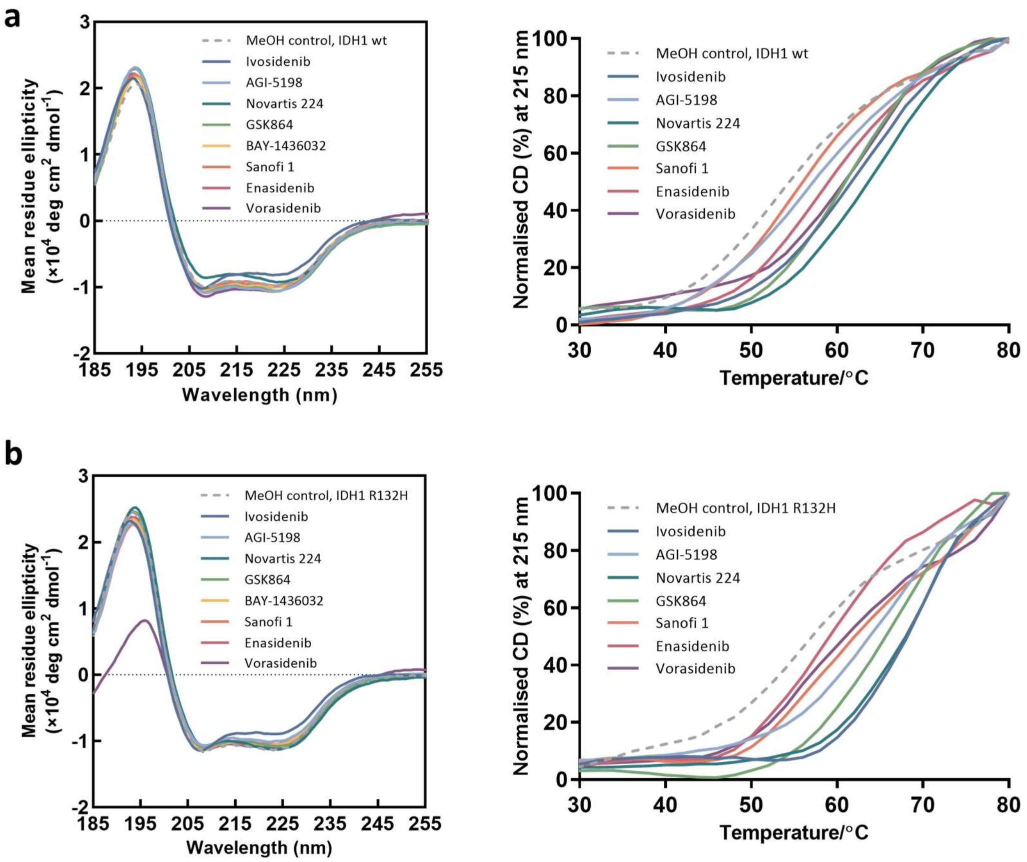

Figure S5. CD spectra of wt IDH1 and IDH1 R132H (0.2 mg/mL, 4.2 µM) with mIDH1/2 inhibitors (42 µM), in 10 mM sodium phosphate, pH 7.5. CD measurements at a single temperature were performed at 20 °C over 185–260 nm. T_m determinations were performed at 215 nm over 10–80 °C. Data are averaged traces of 3 technical replicates. See the Experimental Section for details.

a. CD spectra of wt IDH1 with mIDH1/2 inhibitors (Ivosidenib, AGI-5198, Novartis 224, GSK864, BAY-1436032, Sanofi 1, Enasidenib, Vorasidenib). wt IDH1 with 0.42% (v/v) MeOH was measured as a control. No substantial difference was observed with or without inhibitors over 185–260 nm at 20 °C. T_m of wt IDH1 with inhibitors showed that all the tested inhibitors stabilize wt IDH1, with Novartis 224 causing the greatest T_m shift of 9.7 °C.

b. CD spectra of IDH1 R132H with mIDH1/2 inhibitors (Ivosidenib, AGI-5198, Novartis 224, GSK864, BAY-1436032, Sanofi 1, Enasidenib, Vorasidenib). IDH1 R132H with 0.42% (v/v) MeOH was measured as a control. No substantial difference was observed with and without inhibitors over 185–260 nm at 20 °C, except for Vorasidenib which caused low mean residue ellipticity (MRE) below 200 nm. T_m of IDH1 R132H with inhibitors showed that all the tested inhibitors stabilize IDH1 R132H, with Ivosidenib and Novartis 224 causing the greatest T_m shift of 11.2 °C.

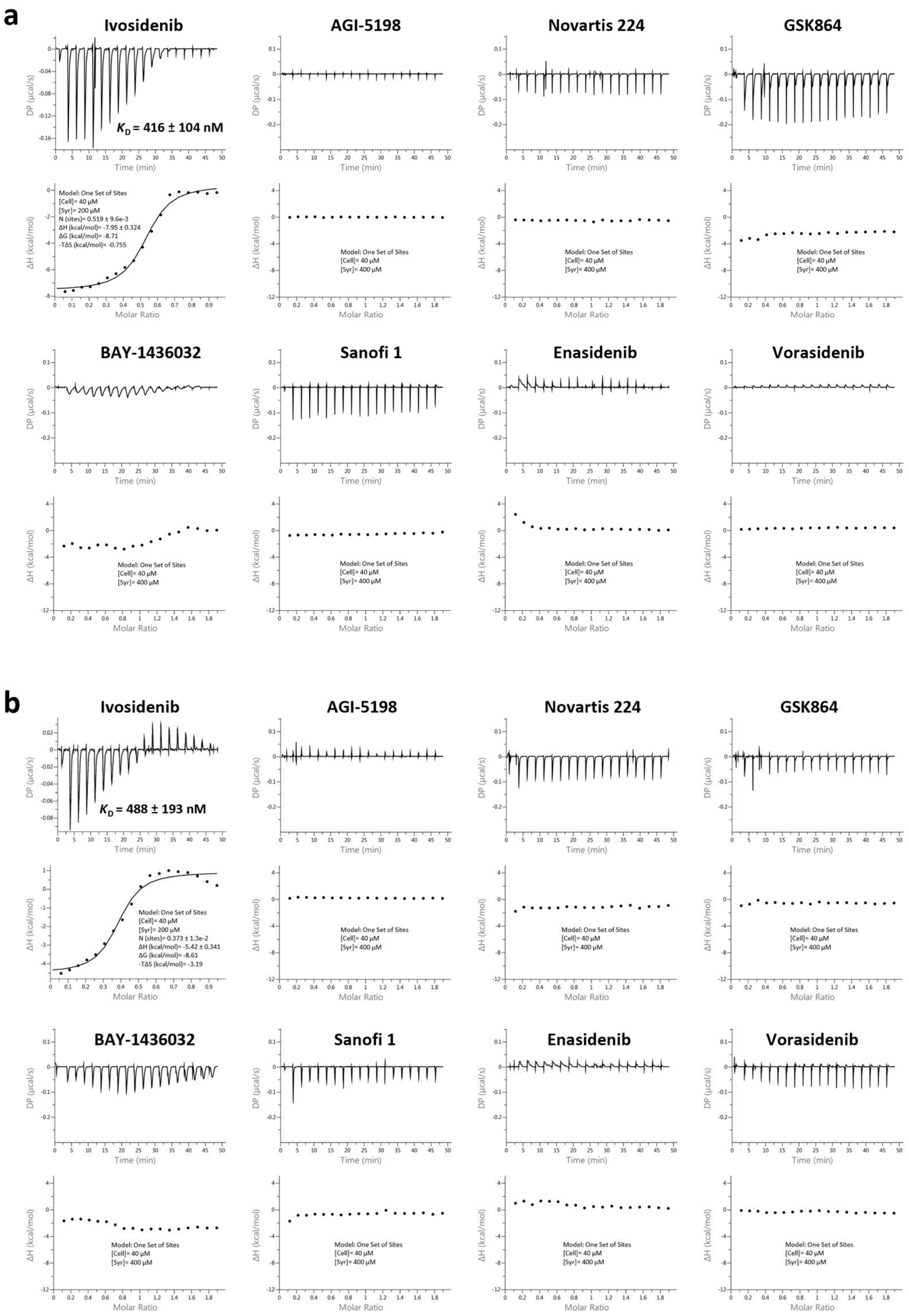

Figure S6. ITC analyses of mIDH1/2 inhibitors titrated against wt IDH1 and IDH1 R132H (40 µM), in 50 mM Tris-HCl, pH 7.5. See the Experimental Section for details.

a. ITC analyses of wt IDH1 (40 µM) with mIDH1/2 inhibitors (400 µM, except 200 µM for Ivosidenib): Ivosidenib, AGI-5198, Novartis 224, GSK864, BAY-1436032, Sanofi 1, Enasidenib, Vorasidenib. No ITC evidence for binding was observed for the inhibitors shown, except for Ivosidenib.
b. ITC analyses of IDH1 R132H (40 µM) with mIDH1/2 inhibitors (400 µM, except 200 µM for Ivosidenib): Ivosidenib, AGI-5198, Novartis 224, GSK864, BAY-1436032, Sanofi 1, Enasidenib, Vorasidenib. No ITC evidence for binding was observed for the inhibitors shown, except for Ivosidenib.

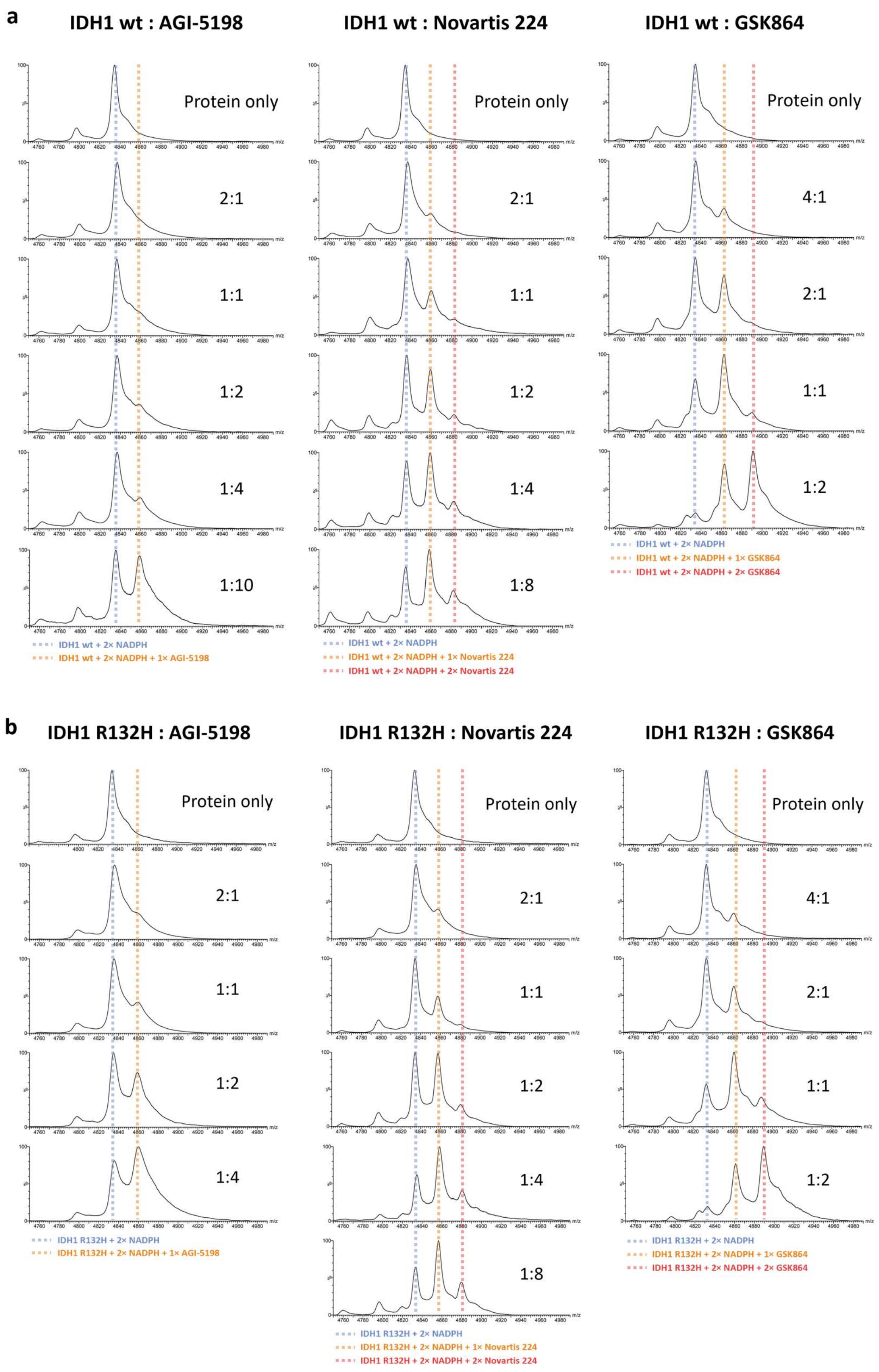

Figure S7. Non-denaturing MS analysis of mIDH1 inhibitors binding to wt IDH1 and IDH1 R132H. Data for the IDH1 dimer m/z = 20^{+} charge state are shown. Cone voltage: 100 V. Buffer: 200 mM ammonium acetate, pH 7.5. See the Experimental Section for details. Inhibitors were titrated in two-fold step changes against IDH1 (50 μM) for K_D determinations. The non-denaturing MS results imply a binding stoichiometry of either 1 or 2 inhibitors per IDH1 dimer. Blue dotted line: 2× NADPH bound IDH1 dimer; orange dotted line: 2× NADPH and 1× inhibitor bound IDH1 dimer; red dotted line: 2× NADPH and 2× inhibitors bound IDH1 dimer.

a. Non-denaturing MS analysis of AGI-5198, Novartis 224, GSK864 binding to wt IDH1. AGI-5198 binds wt IDH1 with a stoichiometry of 1 inhibitor per IDH1 dimer. Novartis 224 and GSK864 bind wt IDH1 with an observed stoichiometry of 2 inhibitors per IDH1 dimer.

b. Non-denaturing MS analysis of AGI-5198, Novartis 224, GSK864 binding to IDH1 R132H. Similarly to the results for wt IDH1, AGI-5198 binds IDH1 R132H with a stoichiometry of 1 inhibitor per IDH1 dimer. Novartis 224 and GSK864 bind IDH1 R132H with an observed stoichiometry of 2 inhibitors per IDH1 dimer.

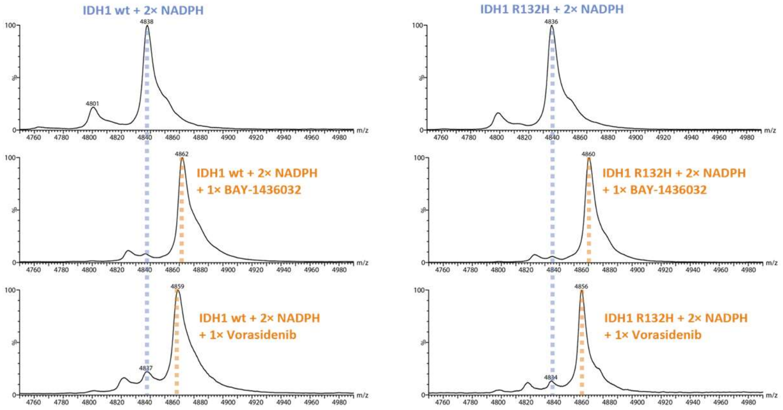

Figure S8. Non-denaturing MS analysis of BAY-1436032 and Vorasidenib binding to wt IDH1 and IDH1 R132H. Data for the IDH1 dimer m/z = 20^+ charge state are shown. Cone voltage: 100 V. Buffer: 200 mM ammonium acetate, pH 7.5. See the Experimental Section for details. The results imply that both BAY-1436032 and Vorasidenib (200 μM) bind wt IDH1 and IDH1 R132H (50 μM) with a stoichiometry of 1 inhibitor per IDH1 dimer. Blue dotted line: 2× NADPH bound IDH1 dimer; orange dotted line: 2× NADPH and 1× inhibitor bound IDH1 dimer.

Note that further work is required to define the dynamic motions involved in the mIDH allosteric inhibition mechanisms, as is the case for conformational changes during IDH catalysis, including with how the inhibitors bind to different E.S/E.intermediate/E.P complexes. The motions may differ for different inhibitor series, some of which bind with a stoichiometry of 1 inhibitor per IDH dimer and some with a stoichiometry of 2 inhibitors per IDH dimer, as shown by non-denaturing MS studies. Note that our binding studies were carried out with IDH1 R132H which copurifies with two NADPH molecules, which is not necessarily the catalytically active form[14]. The subtle, but effective nature of allosteric inhibition of mIDH, involving dynamic conformational changes, raises the question of whether rather than inhibiting mIDH, it may be possible, to identify compounds that either restore wildtype reactions or promote new therapeutically useful reactions, perhaps by employing high throughput screens under appropriate conditions or by derivatisation of known allosterically binding inhibitor scaffolds.

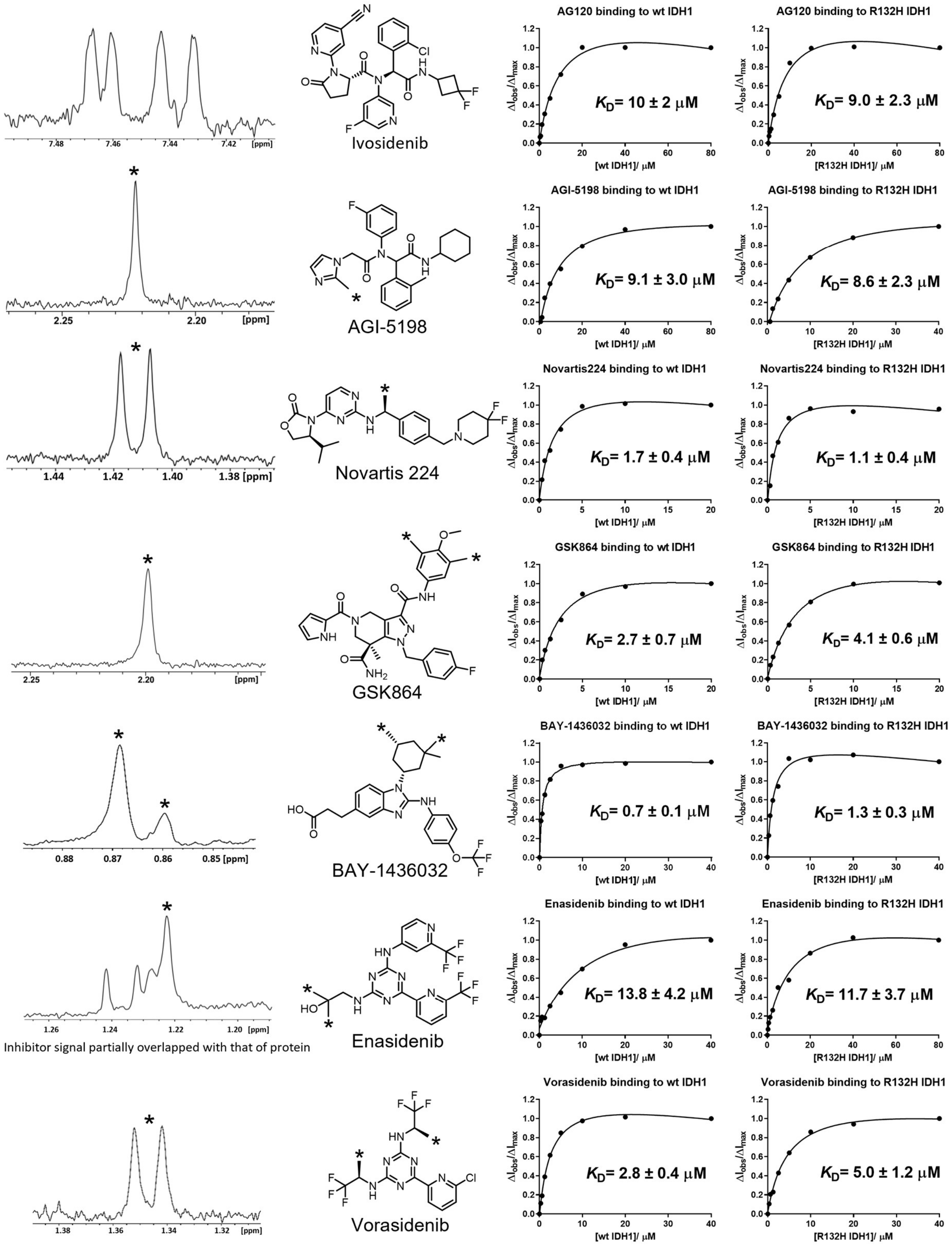

Figure S9. K_Ds of mIDH1 inhibitors with wt IDH1 and IDH1 R132H in 50 mM Tris-D_{11}-HCl, pH 7.5 in 90% H_2O/10% D_2O (v/v), as measured by ^{1}H NMR (CPMG-edited, 700 MHz) spectroscopy. See the Experimental Section for details. IDH1 was titrated against 10 μM inhibitor (Ivosidenib, AGI-5198, Novartis 224, GSK864, BAY-1436032, Enasidenib, Vorasidenib). The areas of the inhibitor peak(s) shown were integrated for different protein concentrations. Note that for Enasidenib, the inhibitor signals partially overlap with the protein signals. Graphs of $\Delta I_{obs}/\Delta I_{max}$ against [protein]/μM were fitted using GraphPad Prism to obtain K_D. $\Delta I_{obs}/\Delta I_{max}=(I_{max}-I_{obs})/(I_{max}-I_{min})$ where I_{max} is the maximum inhibitor peak integral observed when no protein is added, I_{obs} is the observed inhibitor peak integral at a particular protein concentration, and I_{min} is the minimum inhibitor peak integral (typically ~0) when the binding is fully saturated. All inhibitors tested bind both wt IDH1 and IDH1 R132H with similar affinities.

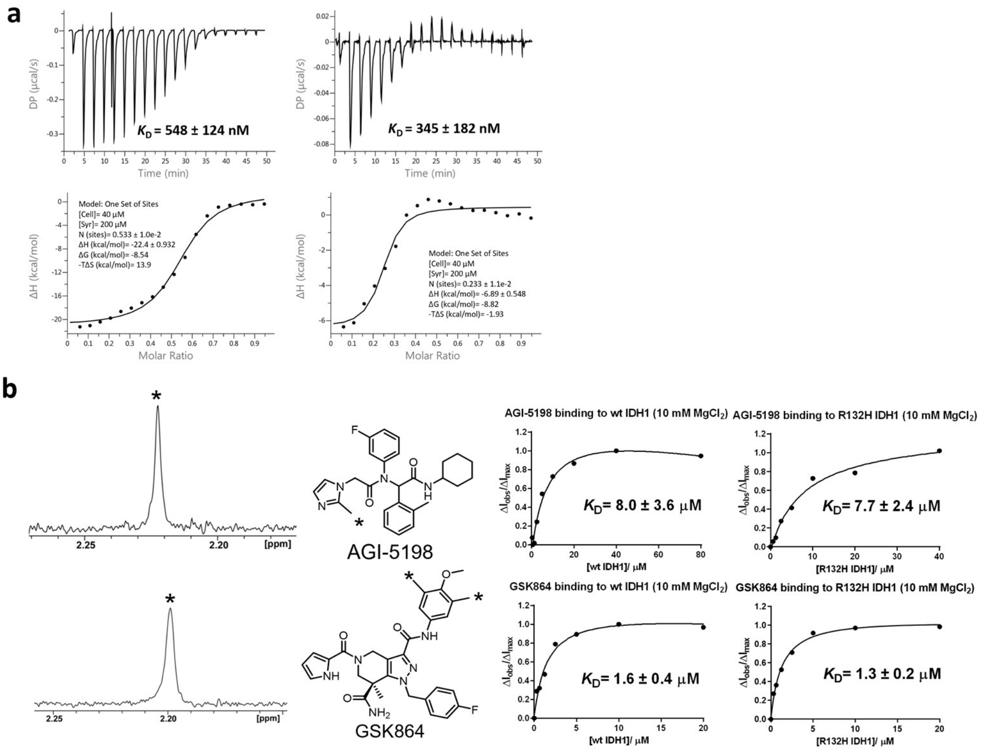

Figure S10. The presence of $MgCl_2$ has no observable effect on inhibitor binding to wt IDH1 and IDH1 R132H, as measured by ITC and ^1H NMR (CPMG-edited, 700 MHz) spectroscopy. See the Experimental Section for details.

a. ITC analyses of wt IDH1 and IDH1 R132H (40 µM) with Ivosidenib (200 µM) in 50 mM Tris-HCl, pH 7.5, 5 mM $MgCl_2$. The K_Ds obtained are similar to those without $MgCl_2$ (**Figure 4b**).
b. K_D of AGI-5198 and GSK864 with wt IDH1 and IDH1 R132H in 50 mM Tris-D_{11}-HCl, pH 7.5, 10 mM $MgCl_2$ in 90% H_2O/10% D_2O (v/v), as determined by ^1H NMR (CPMG-edited, 700 MHz) spectroscopy. The K_Ds obtained are similar to those obtained without $MgCl_2$ (**Figure S9**).

SUPPLEMENTARY REFERENCES

(1) Wang, F.; Travins, J.; DeLaBarre, B.; Penard-Lacronique, V.; Schalm, S.; Hansen, E.; Straley, K.; Kernytsky, A.; Liu, W.; Gliser, C.; Yang, H.; Gross, S.; Artin, E.; Saada, V.; Mylonas, E.; Quivoron, C.; Popovici-Muller, J.; Saunders, J. O.; Salituro, F. G.; Yan, S.; Murray, S.; Wei, W.; Gao, Y.; Dang, L.; Dorsch, M.; Agresta, S.; Schenkein, D. P.; Biller, S. A.; Su, S. M.; de Botton, S.; Yen, K. E. Targeted Inhibition of Mutant IDH2 in Leukemia Cells Induces Cellular Differentiation. *Science* **2013**, *340* (6132), 622–626. https://doi.org/10.1126/science.1234769.

(2) Yen, K.; Travins, J.; Wang, F.; David, M. D.; Artin, E.; Straley, K.; Padyana, A.; Gross, S.; DeLaBarre, B.; Tobin, E.; Chen, Y.; Nagaraja, R.; Choe, S.; Jin, L.; Konteatis, Z.; Cianchetta, G.; Saunders, J. O.; Salituro, F. G.; Quivoron, C.; Opolon, P.; Bawa, O.; Saada, V.; Paci, A.; Broutin, S.; Bernard, O. A.; de Botton, S.; Marteyn, B. S.; Pilichowska, M.; Xu, Y.; Fang, C.; Jiang, F.; Wei, W.; Jin, S.; Silverman, L.; Liu, W.; Yang, H.; Dang, L.; Dorsch, M.; Penard-Lacronique, V.; Biller, S. A.; Su, S.-S. M. AG-221, a First-in-Class Therapy Targeting Acute Myeloid Leukemia Harboring Oncogenic IDH2 Mutations. *Cancer Discov.* **2017**, 7 (5), 478–493. https://doi.org/10.1158/2159-8290.CD-16-1034.

(3) Ma, R.; Yun, C. H. H. Crystal Structures of Pan-IDH Inhibitor AG-881 in Complex with Mutant Human IDH1 and IDH2. *Biochem. Biophys. Res. Commun.* **2018**, *503* (4), 2912–2917. https://doi.org/10.1016/j.bbrc.2018.08.068.

(4) Popovici-Muller, J.; Lemieux, R. M.; Artin, E.; Saunders, J. O.; Salituro, F. G.; Travins, J.; Cianchetta, G.; Cai, Z.; Zhou, D.; Cui, D.; Chen, P.; Straley, K.; Tobin, E.; Wang, F.; David, M. D.; Penard-Lacronique, V.; Quivoron, C.; Saada, V.; de Botton, S.; Gross, S.; Dang, L.; Yang, H.; Utley, L.; Chen, Y.; Kim, H.; Jin, S.; Gu, Z.; Yao, G.; Luo, Z.; Lv, X.; Fang, C.; Yan, L.; Olaharski, A.; Silverman, L.; Biller, S.; Su, S.-S. M.; Yen, K. Discovery of AG-120 (Ivosidenib): A First-in-Class Mutant IDH1 Inhibitor for the Treatment of IDH1 Mutant Cancers. *ACS Med. Chem. Lett.* **2018**, *9* (4), 300–305. https://doi.org/10.1021/acsmedchemlett.7b00421.

(5) Urban, D. J.; Martinez, N. J.; Davis, M. I.; Brimacombe, K. R.; Cheff, D. M.; Lee, T. D.; Henderson, M. J.; Titus, S. A.; Pragani, R.; Rohde, J. M.; Liu, L.; Fang, Y.; Karavadhi, S.; Shah, P.; Lee, O. W.; Wang, A.; McIver, A.; Zheng, H.; Wang, X.; Xu, X.; Jadhav, A.; Simeonov, A.; Shen, M.; Boxer, M. B.; Hall, M. D. Assessing Inhibitors of Mutant Isocitrate Dehydrogenase Using a Suite of Pre-Clinical Discovery Assays. *Sci. Rep.* **2017**, 7 (1), 12758. https://doi.org/10.1038/s41598-017-12630-x.

(6) Rohle, D.; Popovici-Muller, J.; Palaskas, N.; Turcan, S.; Grommes, C.; Campos, C.; Tsoi, J.; Clark, O.; Oldrini, B.; Komisopoulou, E.; Kunii, K.; Pedraza, A.; Schalm, S.; Silverman, L.; Miller, A.; Wang, F.; Yang, H.; Chen, Y.; Kernytsky, A.; Rosenblum, M. K.; Liu, W.; Biller, S. A.; Su, S. M.; Brennan, C. W.; Chan, T. A.; Graeber, T. G.; Yen, K. E.; Mellinghoff, I. K. An Inhibitor of Mutant IDH1 Delays Growth and Promotes Differentiation of Glioma Cells. *Science (80-.).* **2013**, *340* (6132), 626–630. https://doi.org/10.1126/science.1236062.

(7) Deng, G.; Shen, J.; Yin, M.; McManus, J.; Mathieu, M.; Gee, P.; He, T.; Shi, C.; Bedel, O.; McLean, L. R.; Le-Strat, F.; Zhang, Y.; Marquette, J. P.; Gao, Q.; Zhang, B.; Rak, A.; Hoffmann, D.; Rooney, E.; Vassort, A.; Englaro, W.; Li, Y.; Patel, V.; Adrian, F.; Gross, S.; Wiederschain, D.; Cheng, H.; Licht, S. Selective Inhibition of Mutant Isocitrate Dehydrogenase 1 (IDH1) via Disruption of a Metal Binding Network by an Allosteric Small Molecule. *J. Biol. Chem.* **2015**, *290* (2), 762–774. https://doi.org/10.1074/jbc.M114.608497.

(8) Davis, M. I.; Gross, S.; Shen, M.; Straley, K. S.; Pragani, R.; Lea, W. A.; Popovici-Muller, J.; DeLaBarre, B.; Artin, E.; Thorne, N.; Auld, D. S.; Li, Z.; Dang, L.; Boxer, M. B.; Simeonov, A. Biochemical, Cellular, and Biophysical Characterization of a Potent Inhibitor of Mutant Isocitrate Dehydrogenase IDH1. *J. Biol. Chem.* **2014**, *289* (20), 13717–13725. https://doi.org/10.1074/jbc.M113.511030.

(9) Cho, Y. S.; Levell, J. R.; Toure, B.; Yang, F.; Caferro, T.; Lei, H.; Lenoir, F.; Liu, G.; Palermo, M. G.; Shultz, M. D.; Smith, T.; Costales, A. Q.; Pfister, K. B.; Sendzik, M.; Shafer, C.; Sutton, J.; Zhao, Q. 3-Pyrimidin-4-Yl-Oxazolidin-2-Ones as Inhibitors of Mutant IDH. WO 2013/046136 Al, 2013.

(10) Okoye-Okafor, U. C.; Bartholdy, B.; Cartier, J.; Gao, E. N.; Pietrak, B.; Rendina, A. R.; Rominger, C.; Quinn, C.; Smallwood, A.; Wiggall, K. J.; Reif, A. J.; Schmidt, S. J.; Qi, H.; Zhao, H.; Joberty, G.; Faelth-Savitski, M.; Bantscheff, M.; Drewes, G.; Duraiswami, C.; Brady, P.; Groy, A.; Narayanagari, S.; Antony-Debre, I.; Mitchell, K.; Wang, H. R.; Kao, Y.; Christopeit, M.; Carvajal, L.; Barreyro, L.; Paietta, E.; Makishima, H.; Will, B.; Concha, N.; Adams, N. D.; Schwartz, B.; McCabe, M. T.; Maciejewski, J.; Verma, A.; Steidl, U. New IDH1 Mutant Inhibitors for Treatment of Acute Myeloid

Leukemia. *Nat. Chem. Biol.* **2015**, *11* (11), 878–886. https://doi.org/10.1038/nchembio.1930.

(11) Pusch, S.; Krausert, S.; Fischer, V.; Balss, J.; Ott, M.; Schrimpf, D.; Capper, D.; Sahm, F.; Eisel, J.; Beck, A.-C.; Jugold, M.; Eichwald, V.; Kaulfuss, S.; Panknin, O.; Rehwinkel, H.; Zimmermann, K.; Hillig, R. C.; Guenther, J.; Toschi, L.; Neuhaus, R.; Haegebart, A.; Hess-Stumpp, H.; Bauser, M.; Wick, W.; Unterberg, A.; Herold-Mende, C.; Platten, M.; von Deimling, A. Pan-Mutant IDH1 Inhibitor BAY 1436032 for Effective Treatment of IDH1 Mutant Astrocytoma *in Vivo*. *Acta Neuropathol.* **2017**, *133* (4), 629–644. https://doi.org/10.1007/s00401-017-1677-y.

(12) Zheng, B.; Yao, Y.; Liu, Z.; Deng, L.; Anglin, J. L.; Jiang, H.; Prasad, B. V. V.; Song, Y. Crystallographic Investigation and Selective Inhibition of Mutant Isocitrate Dehydrogenase. *ACS Med. Chem. Lett.* **2013**, *4* (6), 542–546. https://doi.org/10.1021/ml400036z.

(13) Konteatis, Z.; Artin, E.; Nicolay, B.; Straley, K.; Padyana, A. K.; Jin, L.; Chen, Y.; Narayaraswamy, R.; Tong, S.; Wang, F.; Zhou, D.; Cui, D.; Cai, Z.; Luo, Z.; Fang, C.; Tang, H.; Lv, X.; Nagaraja, R.; Yang, H.; Su, S. M. S.-S. M. S. M.; Sui, Z.; Dang, L.; Yen, K.; Popovici-Muller, J.; Codega, P.; Campos, C.; Mellinghoff, I. K.; Biller, S. A. Vorasidenib (AG-881): A First-in-Class, Brain-Penetrant Dual Inhibitor of Mutant IDH1 and 2 for Treatment of Glioma. *ACS Med. Chem. Lett.* **2020**, *11* (2), 101–107. https://doi.org/10.1021/acsmedchemlett.9b00509.

(14) Roman, J. V; Melkonian, T. R.; Silvaggi, N. R.; Moran, G. R. Transient-State Analysis of Human Isocitrate Dehydrogenase I: Accounting for the Interconversion of Active and Non-Active Conformational States. *Biochemistry* **2019**, *58* (52), 5366–5380. https://doi.org/10.1021/acs.biochem.9b00518.
